# An ocean front dataset for the Mediterranean sea and southwest Indian ocean

**DOI:** 10.1038/s41597-023-02615-z

**Published:** 2023-10-21

**Authors:** Floriane Sudre, Ismael Hernández-Carrasco, Camille Mazoyer, Joel Sudre, Boris Dewitte, Véronique Garçon, Vincent Rossi

**Affiliations:** 1 Aix Marseille Université, Université de Toulon, CNRS, IRD, MIO, Marseille, France; 2 Mediterranean Institute for Advanced Studies (UIB-CSIC), Miquel Marques, 21, Esporles, 07190 Balearic Islands, Spain; 3 UAR 2013 CPST, IR DATA TERRA, Z.P. de Brégaillon - CS 20330, 83507 Montpellier, La Seyne Sur Mer France; 4 Centro de Estudios Avanzados en Zonas Aridas, Facultad de Ciencias del Mar, Universidad Catolica del Norte, Coquimbo, Chile; 5 Departamento de Biología Marina, Universidad Católica del Norte, Coquimbo, Chile; 6 Center for Ecology and Sustainable Management of Oceanic Islands (ESMOI), Faculty of Marine Sciences, Catholic University of the North, Coquimbo, Chile; 7 UMR5318 Climat, Environnement, Couplages et Incertitudes (CECI), Toulouse, France; 8 University of Liège, Liège, Belgium

**Keywords:** Physical oceanography, Physical oceanography

## Abstract

Fronts are ubiquitous discrete features of the global ocean often associated with enhanced vertical velocities, in turn boosting primary production. Fronts thus form dynamical and ephemeral ecosystems where numerous species meet across all trophic levels. Fronts are also targeted by fisheries. Capturing ocean fronts and studying their long-term variability in relation with climate change is thus key for marine resource management and spatial planning. The Mediterranean Sea and the Southwest Indian Ocean are natural laboratories to study front-marine life interactions due to their energetic flow at sub-to-mesoscales, high biodiversity (including endemic and endangered species) and numerous conservation initiatives. Based on remotely-sensed Sea Surface Temperature and Height, we compute thermal fronts (2003–2020) and attracting Lagrangian coherent structures (1994–2020), in both regions over several decades. We advocate for the combined use of both thermal fronts and attracting Lagrangian coherent structures to study front-marine life interactions. The resulting front dataset differs from other alternatives by its high spatio-temporal resolution, long time coverage, and relevant thresholds defined for ecological provinces.

## Background & Summary

Fronts are ubiquitous dynamical features of both coastal and open ocean. This is where water masses of different properties converge, thereby enhancing nutrient availability and local primary productivity^1–3^. Ocean fronts thus form ephemeral ecosystems where numerous species gather^4–6^: from phytoplankton communities^7,8^, all the way up the food chain to top predators such as sea birds^9,10^, tuna^11,12^ and marine mammals^13,14^.

Numerous techniques have been developed to define and identify ocean fronts. Front detection methods most commonly used by the scientific community rely on the horizontal distribution of oceanic tracers (temperature, salinity, nutrients, etc.) and are either gradient-based^15,16^ or histogram-based^17,18^, while others make use of entropy^19^, neural networks^20^ or singularity exponents^21,22^. While remotely-sensed chlorophyll-a and salinity could be studied, satellite observations of sea surface temperature (SST) provide the most easily-accessible and longest input dataset for front detection^23,24^. Additionally thermal fronts are usually highly spatially correlated to density and chlorophyll-a fronts as well^11,25,26^. Subsequently, SST observations are well-suited to Eulerian front diagnostics and to study front-marine life interactions. Histogram-based front detection, such as the Cayulla and Cornillon algorithm^17^ which was further improved by Nieto *et al*.^18^, is the most widely used method to detect persistent front edges on large spatial scales from satellite SST observations^11,12,23^. However, gradient-based methods present significant advantages to the study of regional fronts dynamics compared to histogram-based methods^6^. They detect local fronts when the gradient of a specific property (temperature, salinity, nutrient, sea surface height, etc.) exceeds a pre-defined threshold. Gradient-based methods, such as the Belkin and O’Reilly Algorithm (BOA)^16^ used in this study, thus provide a gradient magnitude instead of a simple binary edge, require less parameter fine-tuning, and thus better reveal region-specific dynamics and variability^6,27^. The BOA improves on the original Canny Algorithm^15^ by reducing gradient-generated noise while preserving the shape of frontal structures^16^ and it has been successfully applied to the South China Sea^28^, the East China Sea^29^, the Kuroshio current^30^ and the Mozambique Channel^27^.

While the Eulerian perspective brought by thermal gradients describes well the spatial features of the flow at a given time, the Lagrangian dynamical approach has the added benefit of integrating both spatial and temporal current variability and brings additional information about transport and mixing properties of the fluid. In particular, Finite-size Lyapunov exponents (FSLEs)^31^ used in this study are well-suited to evaluate horizontal mixing and transport in geophysical flows^7,32,33^. High FSLE values reveal lines acting as transport barriers, also expressed as hyperbolic Lagrangian coherent structures (LCS), which help to identify filaments, fronts or eddy boundaries. Ridges of backward-in-time FSLE (also referred to as “backward FSLEs”) fields characterize regions of locally high convergence of particle trajectories (attracting LCS) while forward-in-time FSLEs identify regions of locally high divergence (repelling LCS). These dynamical structures greatly organize the fluid motion around them, providing a flow mapping with the main routes of transport^34,35^. Here we provide backward FSLEs to unveil attracting LCS. Tracers (such as temperature, nutrients, pollutants, etc.) converge and spread along attracting LCS, creating filament-like structures of high tracer concentration. This property offers a direct and clear physical interpretation of the attracting LCS.

FSLEs are complementary to thermal gradients because they are not vulnerable to cloud cover, they are able to reveal dynamics at a smaller scale than the resolution of the velocity field^34^, and they provide useful transport diagnosis of tracers^7,36^. Additionally, FSLEs are especially useful to study marine bio-physical interactions. Indeed, the LCS revealed by the FSLE field separate regions with different dynamics and often coincide with sharp tracer gradients^37^ so that they can be interpreted as dynamical fronts. FSLEs have been used to understand the dynamics of tracers like SST and chlorophyll-a^7,8,38,39^, which have direct implications on the behavior of marine life across all trophic levels^5,10,13,14,40–42^.

**Fig. 1:**
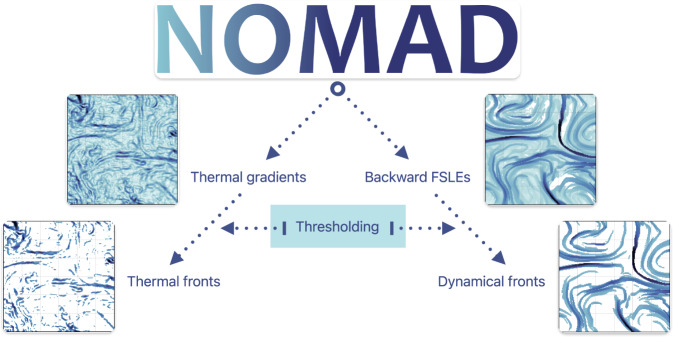
Illustration showing how NOMAD^51^ users can go from thermal gradients (resp. backward FSLEs) to thermal (resp. dynamical) fronts via region-specific thresholding, with example of fields before and after thresholding. All fields were taken on July 1st, 2020. The colormap is from cmocean^80^ and with the same range as in Fig. 2.

The present dataset offers the synergy of both Eulerian and Lagrangian perspectives by providing a multi-decadal record of both thermal gradient and backward FSLE fields at high resolution. Thermal gradients (resp. backward FSLEs) fields are computed on a 1 km (resp. 1.56 km) grid, covering the period 2003–2020 (resp. 1994–2020), for two oceanic regions characterized by high biodiversity under conservation concerns: the Mediterranean Sea (MedSea) and the Southwest Indian Ocean (SWIO). Thermal gradients (resp. backward FSLEs) lead to thermal (resp. dynamical) fronts after region-appropriate thresholding, as illustrated in Fig. 1.

Other data products informing on ocean fronts have been developed. Belkin *et al*.^23^ produced a 9 km-resolution persistent front database for major Large Marine Ecosystems^43^. Additionally, daily backward FSLEs^44^ (accessed on Jan 20th 2023) have been computed from altimetry derived daily velocities on a 4 km global grid since 1994 following the method of d’Ovidio *et al*.^32^. Finally, the BOA has been used by the National Oceanic and Atmospheric Administration (NOAA) since 2013 to produce near real-time chlorophyll-a frontal maps (accessed on Jan 20th, 2023) for the U.S. coastal areas only. Those existing datasets target analyses of much larger spatial scales, their resolution being 2.5 to 9 time coarser than the dataset presented here, and/or do not cover the present geographical domains of interest. Our front dataset differs to those existing by being specifically designed for multidisciplinary research, especially topics linking ocean weather and marine ecology^45^. It is dedicated to researchers who investigate front dynamics at space and time scales that relate to the highly patchy distribution of biological production and the rapidly evolving behavior (for foraging, mating, socializing, migrating, etc.) of marine species, especially top marine predators. Additionally, the greater length of the provided time series allows for analyzing long-term variability and trends. Our ocean front dataset for the Mediterranean Sea and the Southwest Indian Ocean aims to bridge the gap between advances in the identification of physical oceanic features and the search of general rules governing how marine life across trophic levels exploits the highly heterogeneous seascape.

## Methods

### Thermal gradients

We use the BOA^16^, and more specifically Benjamin Galuardi’s pseudo-code written in R (available on https://github.com/galuardi/boaR, accessed on May 19th, 2022) to compute thermal gradient magnitudes. We apply the BOA on daily satellite Sea Surface Temperatures (SST) from the Multi-scale Ultra-high Resolution sea-surface temperature analysis (MUR SST, accessed on Jan 10th, 2023)^46^, the resulting field is a thermal gradient expressed in °C/km. The resulting Southwestern Indian Ocean (SWIO) thermal gradient dataset covers latitudes between 5°S and 35°S and longitudes between 30°E to 80°E, and the Mediterranean Sea (MedSea) dataset covers latitudes between 30°N and 46°N and longitudes between −6°E to 37°E. The outputs for both geographical domains are produced daily from 2003 to 2020 and at the same spatial resolution of 1/100° which is about 1 km.

Each daily thermal gradient map includes a flag variable indicating whether a given pixel is located on land (flag = 2), or if it is “suitable” (flag = 0) or “unsuitable” (flag = 1) for thermal front analysis. Pixels are deemed “unsuitable” based on MUR SST standard deviation of the formal estimation error, which is considered to be a good estimate on MUR analysis uncertainty^46^. When the MUR SST standard deviation of the estimation error is high, the thermal gradient field is smoothed out and possible front or eddy structures are obfuscated. We thus recommend applying the flag before any further analysis of the thermal gradient field. We set the MUR SST standard deviation of the estimation error threshold, i.e. threshold above which a pixel is deemed “unsuitable” for front analysis, at 0.0384 °C. The choice of threshold is based on a preliminary comparison between MUR SST standard deviation and our thermal gradient dataset.

### Finite-size Lyapunov exponents

In this work, backward-in-time FSLEs in the SWIO are computed from remotely-sensed Sea Surface Height (SSH) at 1/4° spatial resolution from Global Total Surface and 15 m Current (CLS, 2018, COPERNICUS-GLOBCURRENT, accessed on Jan 10th, 2023) from Altimetric absolute geostrophic velocities and Modeled Ekman Current Reprocessing^47^. In the MedSea, backward FSLEs are computed from daily absolute geostrophic surface currents derived from Sea Level Anomalies (SLA) at 1/8° spatial resolution from a SSALTO/DUACS multimission altimeter regional L4 product released in 2016 by AVISO + and based on regional mean dynamic topography^47^ (https://data.marine.copernicus.eu, accessed on Feb 10th, 2023).

Backward FSLEs were obtained following the algorithm described by Hernandez-Carrasco *et al*.^34^. Essentially, the algorithm computes backward FSLEs by integrating backward in time two neighbouring particle trajectories advected in a two-dimensional flow using a fourth order Runge-Kutta scheme with a bilinear interpolation in space and linear in time. The total period of integration is 90 days. The Runge Kutta time step was set at 6 hours to reduce numerical diffusion. As we are interested in simulating fluid particles, we computed trajectories of infinitesimal passive particles. The FSLE value at a given position and time (*x, y, t*) can be expressed as:

$${\rm{FSLE}}\left(x,y,t,{\delta }_{f},{\delta }_{0}\right)=\frac{1}{\tau }\log \left(\frac{{\delta }_{f}}{{\delta }_{0}}\right).$$

**Fig. 2:**
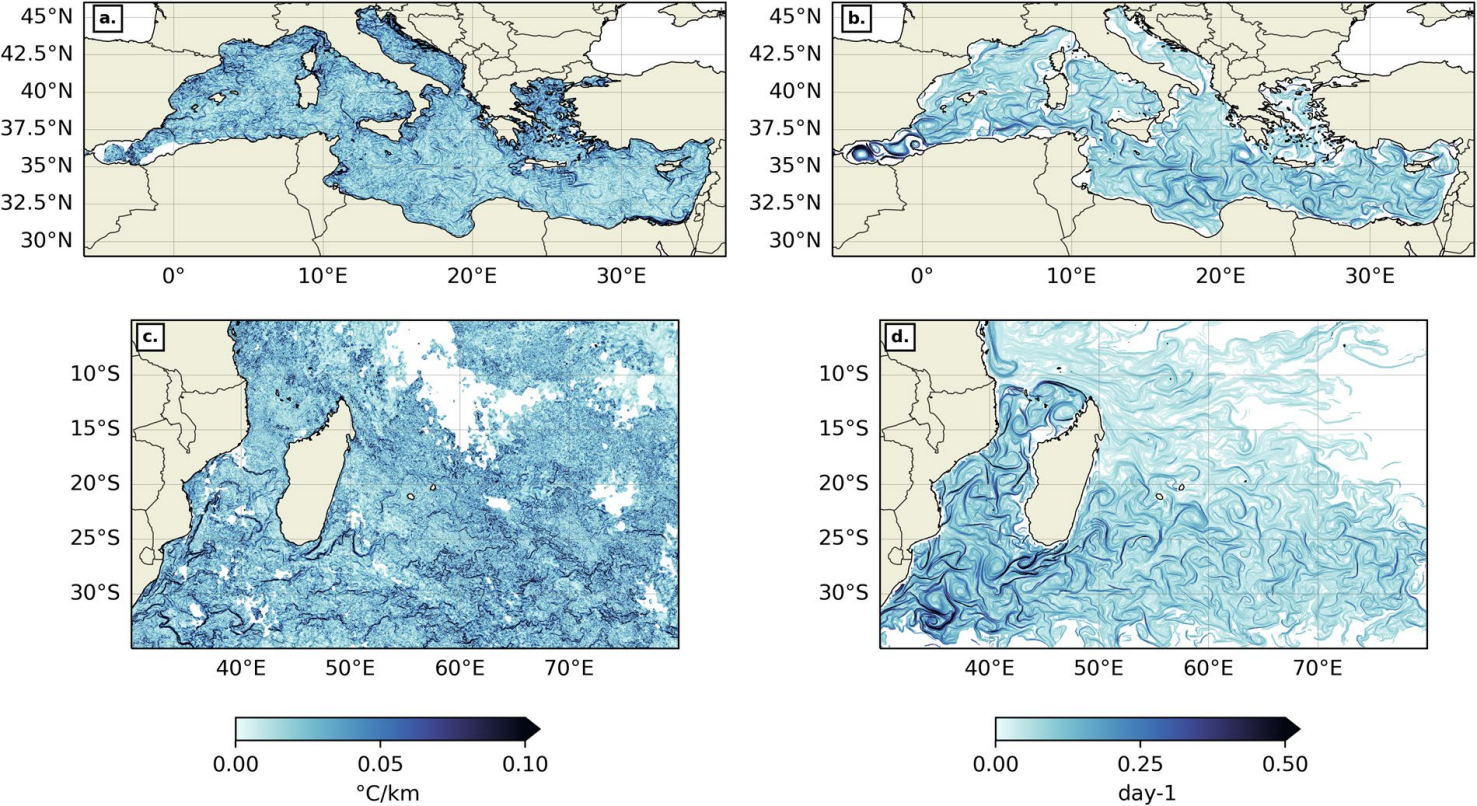
Example snapshots of thermal gradient (**a,c**) and FSLE (**b,d**) fields in the MedSea (**a,b**) and SWIO (**c,d**), for “suitable” pixels only, on July 1st, 2020. The colormap is from cmocean^80^.

*δ*_0_ is the initial distance between a particle at (*x, y, t*) and its 4 closest neighbors. *δ*_0_ corresponds to the spatial resolution of the FSLE grid, which is here 1/64° or approximately 1.56 km. *δ*_*f*_ is the final distance between particles, here it is set so that *δ*_*f*_ = 10*δ*_0_. *τ* is the minimum time (among the 4 particle pairs) that it takes for the particles to reach the distance *δ*_*f*_. FSLEs are thus expressed in day^−1^. Similarly to the thermal front dataset, the SWIO FSLE dataset covers latitudes between 5°S and 35°S and longitude between 30°E to 80°E, and the MedSea dataset covers latitudes between 30°N and 46°N and longitudes between −6°E to 37°E. Both geographical domains have a spatial resolution of 1/64° which is about 1.56 km, and the output is produced daily from 1994 to 2020. Each daily FSLE dataset includes a flag variable indicating whether pixels are located on land (flag = 2), or if they are “suitable” (flag = 0) or “unsuitable” (flag = 1) for front analysis. Pixels are deemed “unsuitable” based on their FSLE value. Pixels whose FSLE value is 0 day^−1^ correspond to particles which have not reached the final distance *δ*_*f*_ after the integration time, i.e. after 90 days. Pixels whose FSLE value is above 900 day^−1^ correspond to beached particles. We thus recommend applying the flag before any further analysis of the FSLE field. Figure 2 is an example snapshot of the thermal gradient and backward FSLE fields in the MedSea and the SWIO.

### Thresholds

**Fig. 3:**
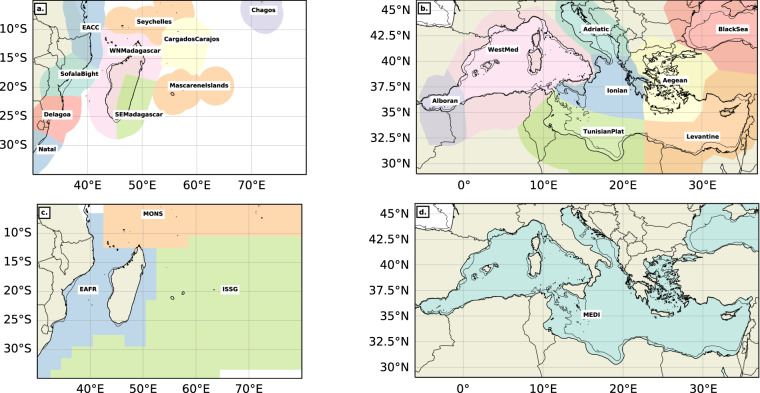
Spalding’s ecoregions^50^ (**a,b**) and Longhurst’s provinces^48,49^ (**c,d**) in the SWIO (**a,c**) and in the MedSea (**b,d**).

One of the advantages of using the BOA and backward FSLEs to detect fronts is that both outputs are continuous fields. Users can then determine the threshold above which thermal gradients or FSLE values belong to a meaningful front, as illustrated in Fig. 1. Since different regions present different ranges of front intensity, that are somehow non-trivially linked to contrasting levels of Eddy-Kinetic Energy^27^, it is relevant to define region-specific thresholds. Moreover, it is very likely that marine organisms aggregate actively along the most adequate fronts for foraging not based on their absolute characteristics but rather on their relative properties (i.e. targeting the most advantageous fronts as compared to all those found in the surroundings). As such, and to adapt our dataset to users interested in the interaction between fronts and marine life at both basin and eco-region scales, we provide thresholds for the MedSea and the SWIO basins but also for each Longhurst’s provinces^48,49^ and Spalding’s ecoregions^50^ falling into each geographical domain (Fig. 3). For both levels of analysis, we set the regional and subregional thresholds at the value of the 70^th^ percentile computed over all the “suitable” pixels in the multidecadal dataset.

### Percentage of “suitable” pixels

The percentage of “suitable” pixels in each region of interest (SPP) is given by:

$${\rm{SPP(region)}}=\frac{{\rm{SPC(region)}}}{{\rm{OPC(region)}}}\ast 100.$$

Where SPC is the number of “suitable” pixels in the region of interest and OPC is the total number of ocean pixels in the same region. Thresholds and SPP are provided for each region and subregion in Table 2 and as attributes in each netcdf file.

## Data Records

The oceaN frOnt dataset for the Mediterranean seA and southwest inDian ocean (NOMAD)^51^ can be accessed through Ifremer online data repository. Figure 2 is a sample snapshot of the thermal gradient and backward-in-time FSLE field in the MedSea and the SWIO. The front dataset is 940 Gb in total, and is separated into 4 folders as detailed in Table 1. Each folder contains yearly subfolders, consisting of daily netcdf files. Each netcdf file provides: thermal gradient or backward FSLE values, pixel quality flags, but also front threshold values specific to each Longhurst’s province and Spalding’s ecoregion found in the MedSea or SWIO, as well as the corresponding percentage of “suitable” pixels found in those same ecologically-relevant subregions of the MedSea and the SWIO. Each netcdf file contains variables as shown below:

**Table 1:** Dataset subfolders details.

Folder name	Region	Variable	size	time period	temporal resolution	spatial resolution
TG_MedSea	MedSea	thermal gradient	128 Gb	2003–2020	daily	1/100° (approx. 1 km)
FSLE_MedSea	MedSea	backward FSLE	61 Gb	1994–2020	daily	1/64° (approx. 1.56 km)
TG_SWIO	SWIO	thermal gradient	507 Gb	2003–2020	daily	1/100° (approx. 1 km)
FSLE_SWIO	SWIO	backward FSLE	244 Gb	1994–2020	daily	1/64° (approx. 1.56 km)

***Thermal gradient netcdf files*** (TG_*region*_*date*.nc)

1. time: date in seconds since 1950-01-01 in the proleptic gregorian calendar.
2. lat: latitude, in decimal degree north.
3. lon: longitude, in decimal degree east.
4. tg: thermal gradient, in °C/km.
5. flag: quality flag, which options are 0 = suitable, 1 = unsuitable, 2 = land.
6. threshold_*subregion*: value of the 70^th^ percentile of the thermal gradient field in a specific subregion, in °C/km.
7. SPP_*subregion*: percentage of pixels deemed suitable in a specific subregion, in %.

***Backward FSLE netcdf files*** (FSLE_*region*_*date*.nc)

1. time: date in seconds since 1950-01-01 in the proleptic gregorian calendar.
2. lat: latitude, in decimal degree north.
3. lon: longitude, in decimal degree east.
4. fsle: backward finite-size Lyapunov exponents, in day^−1^.
5. flag: quality flag, which options are 0 = suitable, 1 = unsuitable, 2 = land.
6. threshold_*subregion*: value of the 70^th^ percentile of the FSLE field in a specific subregion, in day^−1^.
7. SPP_*subregion*: percentage of pixels deemed suitable in a specific subregion, in %.

## Technical Validation

### About thermal fronts

**Fig. 4:**
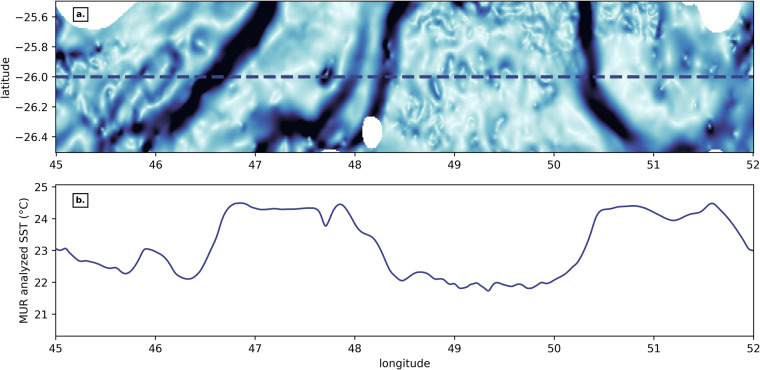
Example of thermal gradient magnitude from the BOA (**a**) and corresponding MUR analysed SST value (**b**) for a section at 26°S in the SWIO, for July 1st, 2020. The colormap of Fig. 4a is from cmocean^80^ and with the same range in °C/km as in Fig. 2.

NOMAD^51^ provides a multidecadal record of thermal gradients, which can be translated into thermal fronts via region-specific thresholding (Fig. 1). The BOA^16^, which we used to compute NOMAD^51^ thermal gradients, has been successfully employed multiple times: in physical oceanography^27,28^, marine ecology^30,52^, as well as by the National Oceanic and Atmospheric Administration (NOAA) since 2013^24^; and can thus be considered as an established method recognized by the research community. Figure 4 illustrates the BOA performance by putting side by side the horizontal thermal gradient issued from the BOA (top panel) and its corresponding MUR SST variation on a section at 26°S. Sharp changes of temperature (ranging approximately from 0.25 to 2 °C in this particular example) coincide with high values of thermal gradients (occurring at spatial scales ranging 10 to 50 km, in this example). High thermal gradient values thus are of the order of several degrees Celsius per 100 km.

#### Choice of SST data

When choosing a suitable SST product, we compromised spatial and temporal resolutions, cloud-contamination, time coverage and public availability. Cloud contamination is a real issue, especially when high spatial resolution is needed: infra-red radiometers cannot sense through cloud cover but microwave sensors do not have a sufficient spatial resolution yet to access small-scales dynamics. We thus opted for the Multi-scale Ultra-high Resolution (MUR) SST analysis^46^, which combines 1 km resolution Moderate Resolution Imaging Spectroradiometer (MODIS) images, to 5–9 km resolution Advanced Very High Resolution Radiometer (AVHRR) products, 25 km resolution micro-wave data, and point-wise *in-situ* SST data. The integration and interpolation of those different satellite products is done by the Multi-Resolution Variational Analysis (MRVA) method, which uses scale-dependent time windows to adjust for various spatial resolutions^46^. MUR analysis thus provides improved spatial coverage and resolution of its SST product compared to conventional L3 data. However, such interpolation procedure also tends to blur oceanic features of interest such as eddies and fronts. It is thus necessary to find a way to identify pixels which have been blurred beyond a certain limit. The MUR SST product comes with a standard deviation of the estimation error (in °C) at each grid point^46^. We use this estimate of MUR analysis uncertainty to identify a suitable threshold above which the multidecadal dataset is considered too blurred for front analysis. The MUR analysis product also provides the time lag to most recent 1 km data, which would help the filtering, but the variable is only included after July 2016. Figure 8 of Sudre *et al*. work^27^ further illustrates the relationship between thermal front detection and the two quality flags available in the MUR dataset. We finally opted for using only the standard deviation of the estimation error to provide a uniform and consistent multidecadal database. Visual comparisons between MUR standard deviation of the estimation error and our thermal gradient dataset were performed to find an arbitrary threshold that would return well-defined frontal structures without too much gaps. A too high estimation error limit would not filter out pixels where the MUR fusion and interpolation procedure blurred fronts and eddies. Yet a too low estimation error limit would lead to many pixels being filtered out, and some front features being hidden despite looking sharp enough to be exploitable. An additional difficulty resides in the fact that the standard deviation of the estimation error is a discrete variable in the MUR SST netcdf files. We found a good compromise for a standard deviation of the estimation error equal to 0.0384 °C.

**Fig. 5:**
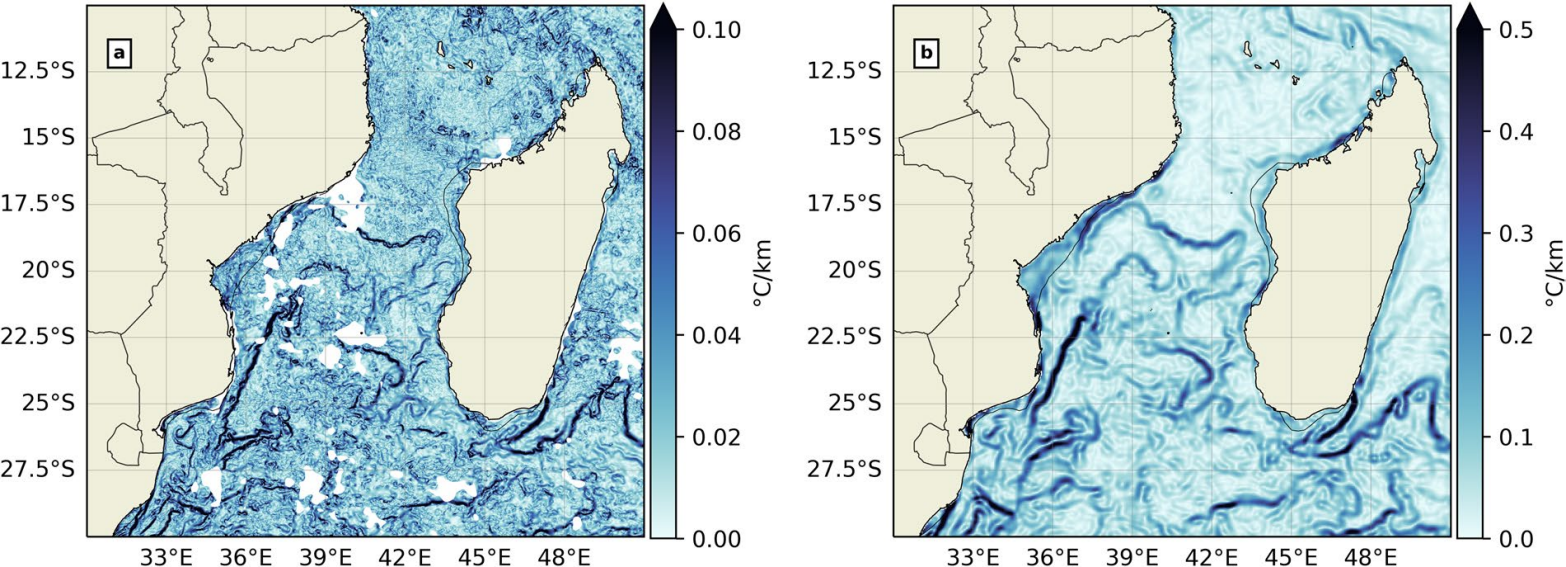
(**a**) thermal gradient output from MUR SST and (**b**) thermal gradient output from SST CCI analysis, in the Mozambique Channel for July 1st, 2020. The colormap is from cmocean^80^.

The European Space Agency Sea Surface Temperature Climate Change Initiative: Analysis product^53^ (SST CCI analysis) could be an attractive alternative to the MUR SST due to its climate-readiness. However, the SST CCI analysis is offered on a 1/20° grid (about 5 km), which is 5 times coarser than that of MUR SST. When pairing with biological observations such as GPS tracks, aerial surveys or sightings, the SST CCI may return spurious and artificial correlations. In designing NOMAD^51^, the authors favored the grid resolution over the climate readiness of the final product to better fit typical scales of marine animal trajectories in the SWIO^54–56^, hence the final choice of SST MUR over the SST CCI analysis. The comparison between both MUR SST and SST CCI analysis is illustrated in Fig. 5 over the front-rich Mozambique Channel^27^. The larger thermal gradient magnitudes from SST CCI could be explained by the contextual median filtering step in the BOA^16^. We hope future ocean front datasets will be able to combine the advantage of both fine-grid resolution and climate-readiness without much compromise.

### About dynamical fronts

NOMAD^51^ provides a multidecadal record of backward-in-time FSLEs, which can be interpreted as dynamical fronts through region-specific thresholding (Fig. 1). In the past 20 years, FSLEs have been recognized by the ocean science community as a reliable way to identify Lagrangian coherent structures^32,34,57–59^. Indeed, FSLEs tend to be better-suited to transport barrier detection than other diagnostics such as the Okubo-Weiss parameter and finite-time Lyapunov exponents^33,59,60^. Additionally, FSLEs perform well in the presence of noise and unresolved scales^34^. Finally FSLEs pairs well with tracer-based front indicators, such as sea-surface temperature^37,61^ and phytoplankton communities^8,42^.

#### Choice of velocity data

**Fig. 6:**
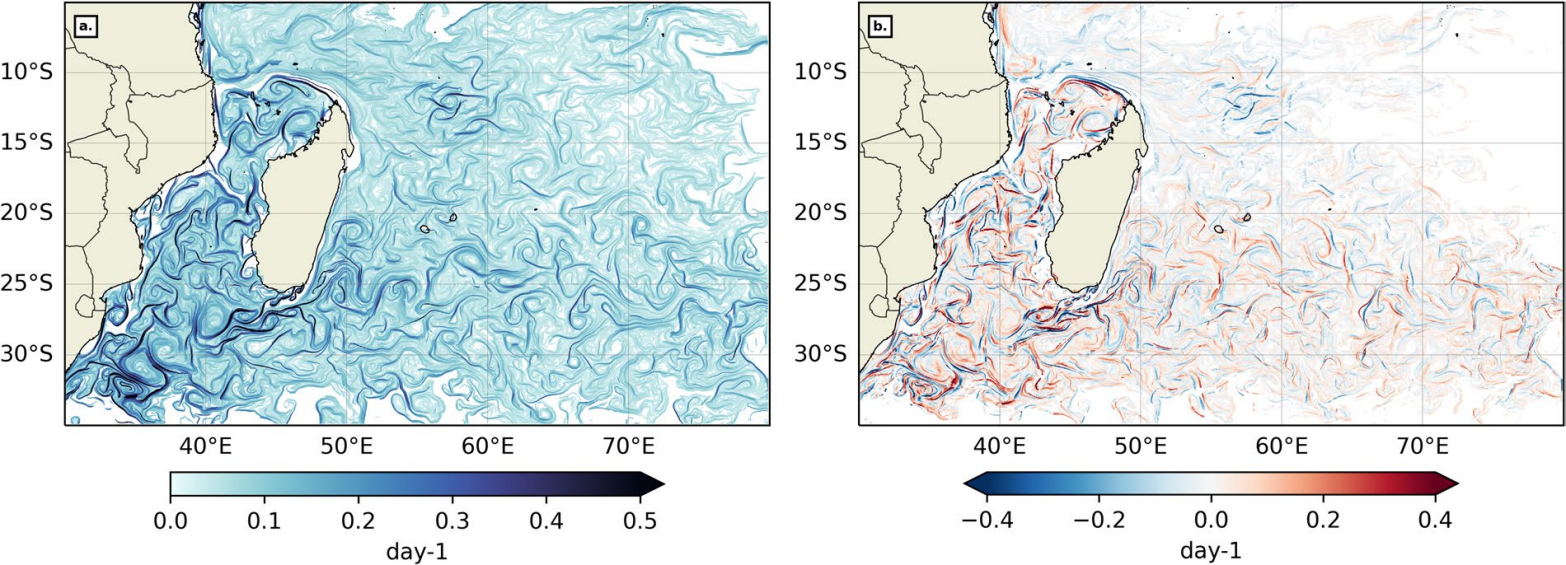
(**a**) Snapshot of FSLEs computed from geostrophy-only currents from the Copernicus Climate Change Service and (**b**) its difference from FSLEs computed from total currents (shown in Fig. 2d), in the SWIO for July 1st, 2020. The colormaps are from cmocean^80^.

The choice of suitable velocity products for the MedSea and the SWIO was motivated by their spatial and temporal resolutions but also the extent of the datasets. The velocity products chosen for the MedSea and SWIO are different because we opted to select the best available product for each domain. Globcurrent 1/4° total velocities^47^ are optimal for the SWIO because of the integration of the Ekman component to the velocity field, its spatial and temporal resolutions and coverage. To the authors knowledge, there is no available operational product providing total velocities (geostrophic + Ekman components) for the MedSea. While the ageostrophic components of the circulation may become relevant in a few rare instances, such as during short-living storms in the Northwestern MedSea^62^, geostrophic currents resolved well the main surface dynamics. We thus opted for the next best: 1/8° geostrophic velocities derived from a SSALTO/DUACS multimission altimeter regional L4 product^47^ for the MedSea. Although both velocity datasets perform well at the mesoscale (typical horizontal scales of 100 km), it is worth noting that they are not climate-ready. The choice of a uniform global climate-ready alternative, such as DUACS geostrophy-only currents distributed by the Copernicus Climate Change Service (C3S) (https://data.marine.copernicus.eu, accessed on Feb 10th, 2023), would have been uniform between both domains but would also have resulted in the loss of the Ekman component in the SWIO and led to a spatial resolution downgrade from 1/8° to 1/4° in the MedSea. The comparison between FSLEs derived from Globcurrent total velocities^47^ and FSLEs derived from geostrophy-only currents from the C3S^63^ in the SWIO is illustrated in Fig. 6. The difference between FSLEs computed from total and geostrophy-only currents reveals small changes in their magnitude and overall pattern, but also show some variations in the positions of the LCS (in the approximate range of 0.5–1°or 50–100 km). These displacements are especially strong in front-rich regions such as the Mozambique Channel and South East of Madagascar. We thus chose to let go of uniformity and climate-readiness to ensure the best rendering of front structures with available altimetry products. Should climate-readiness be of interest to end-users, future front data products could use newer climate-ready products with our proposed methodology.

#### Added-value of spatial resolution

**Fig. 7:**
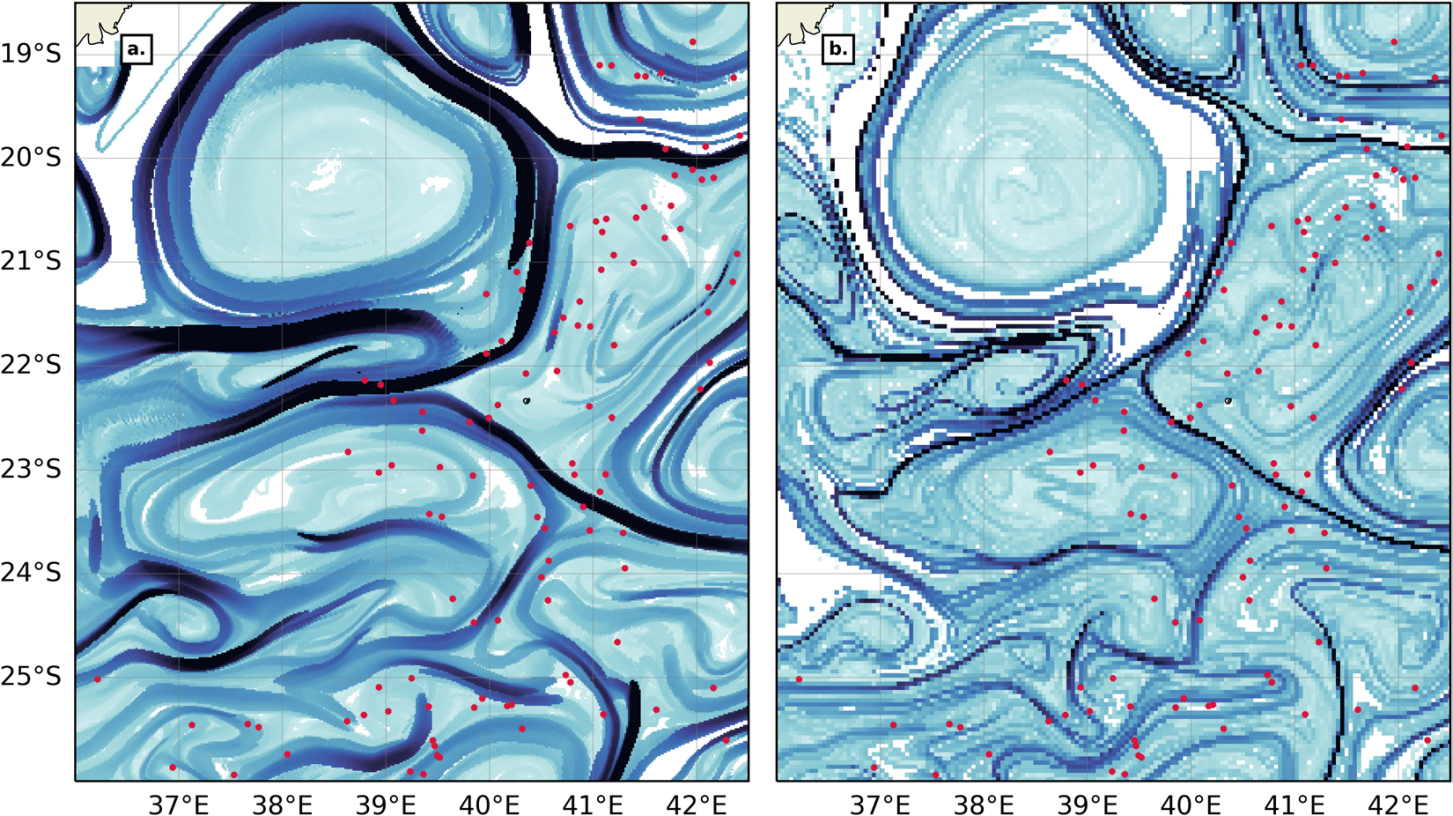
Example of the collocation between randomly-generated points (in red) and FSLE fields for (**a**) NOMAD^51^ backward FSLEs on a 1.5 km grid and for (**b**) AVISO backward near-real-time FSLEs^44^ on a 4 km grid, for Jan 15th, 2020. The colormap is from cmocean^80^ and with the same range as in Fig. 2.

The NOMAD^51^ dataset distinguishes itself from existing front databases by its higher spatial and temporal resolutions. A great advantage of FSLEs is that they are robust to small changes in the velocity field, and they also exhibit typical multifractal properties^34^. These properties ensure that FSLEs produced on a finer-grid than the original velocity grid are indeed meaningful and not artificial. They cannot resolve all the small scales processes, but they render well the dynamics driven by the coarser velocity field. We argue that spatial resolution is critical when investigating fronts-marine life interactions. To illustrate this statement, we compare NOMAD^51^ backward FSLE dataset to the AVISO FSLE product^44^ in the SWIO. The AVISO FSLE field is computed from 1/4° SSALTO/Duacs delayed-time global ocean absolute geostrophic currents (DUACS2018 DT MADT UV products) on a 4 km grid. The AVISO FSLE product^44^ differs from NOMAD^51^ FSLEs in several parameters including velocity field, spatial resolution, times of integration, *δ*_0_ and *δ*_*f*_. We have already contrasted the difference between total and geostrophy-only currents in FSLE computations in the previous paragraph. Additionally, we assume that the AVISO FSLE product time of integration, *δ*_0_ and *δ*_*f*_ have been optimized for the flow field. We will thus only focus here on why we think the higher spatial resolution of the NOMAD^51^ FSLE field is a significant added-value for biological use. We generated 1000 series of 100 to 200 points. Each point is separated from the next by a random direction and a distance of the order of 50–100 km, consistent with typical scales of marine animal presence/absence data (including sightings, GPS tracks, aerial survey, etc.), like those of whale sharks^54^, sea turtles^55^ or the great frigatebirds^56^ in the SWIO. The next step is to collocate each series of points with both NOMAD and AVISO FSLE fields^44^, taken on the same date. For each series the date was picked randomly in the year 2020 to avoid any bias brought on by seasonality. Figure 7 is an example snapshot of NOMAD^51^ and AVISO^44^ FSLE fields overlapped by a sample series of randomly-generated points in the South Mozambique Channel. Lastly, for each of the 1000 series, and relating to both finer NOMAD^51^ FSLE (1.56 km resolution) and coarser AVISO^44^ FSLE (4 km resolution) fields, we extracted 3 metrics:

- the mean FSLE value located under each randomly-generated point,
- the percentage of points collocated with FSLE front pixels (defined by subregional thresholds as in Table 2),

**Table 2:** Thresholds and SPP for each region, Longhurst’s provinces (LH)^48,49^ and Spalding’s Marine Ecoregions of the World (MEOW)^50^.

Region	Classification	Subregion	Thermal Gradients (°C/km)		FSLEs (day^−1^)	
			Threshold	SPP	Threshold	SPP
MedSea	WHOLE	n/a	0.033	83.8%	0.123	57.3%
MedSea	LH	MEDI	0.033	83.6%	0.123	59.8%
MedSea	MEOW	Adriatic Sea	0.039	78.0%	0.086	44.0%
MedSea	MEOW	Aegean Sea	0.039	78.9%	0.135	49.0%
MedSea	MEOW	Alboran Sea	0.039	75.9%	0.254	41.3%
MedSea	MEOW	Ionian Sea	0.031	84.4%	0.113	73.3%
MedSea	MEOW	Levantine Sea	0.032	86.4%	0.142	75.6%
MedSea	MEOW	Tunisian Plateau	0.030	85.9%	0.111	76.4%
MedSea	MEOW	Western Mediterranean	0.033	85.6%	0.119	69.3%
SWIO	WHOLE	n/a	0.035	74.1%	0.105	73.3%
SWIO	LH	EAFR	0.036	78.5%	0.166	89.3%
SWIO	LH	ISSG	0.036	77.1%	0.094	74.7%
SWIO	LH	MONS	0.034	60.3%	0.083	56.3%
SWIO	MEOW	Bight of Sofala	0.033	79.2%	0.190	90.4%
SWIO	MEOW	Cargados Carajos	0.034	68.3%	0.077	77.0%
SWIO	MEOW	Chagos	0.036	49.7%	0.075	42.7%
SWIO	MEOW	Delagoa	0.038	83.3%	0.179	95.2%
SWIO	MEOW	East African Coral Coast	0.032	71.7%	0.128	79.0%
SWIO	MEOW	Mascarene Islands	0.033	81.1%	0.089	88.0%
SWIO	MEOW	Natal	0.041	76.5%	0.210	89.0%
SWIO	MEOW	Seychelles	0.034	67.5%	0.083	66.3%
SWIO	MEOW	Southeast Madagascar	0.037	76.6%	0.142	93.7%
SWIO	MEOW	Western and Northern Madagascar	0.034	75.8%	0.146	86.6%
- and the mean distance between each point and its nearest FSLE front (defined by subregional thresholds as in Table 2)

**Fig. 8:**
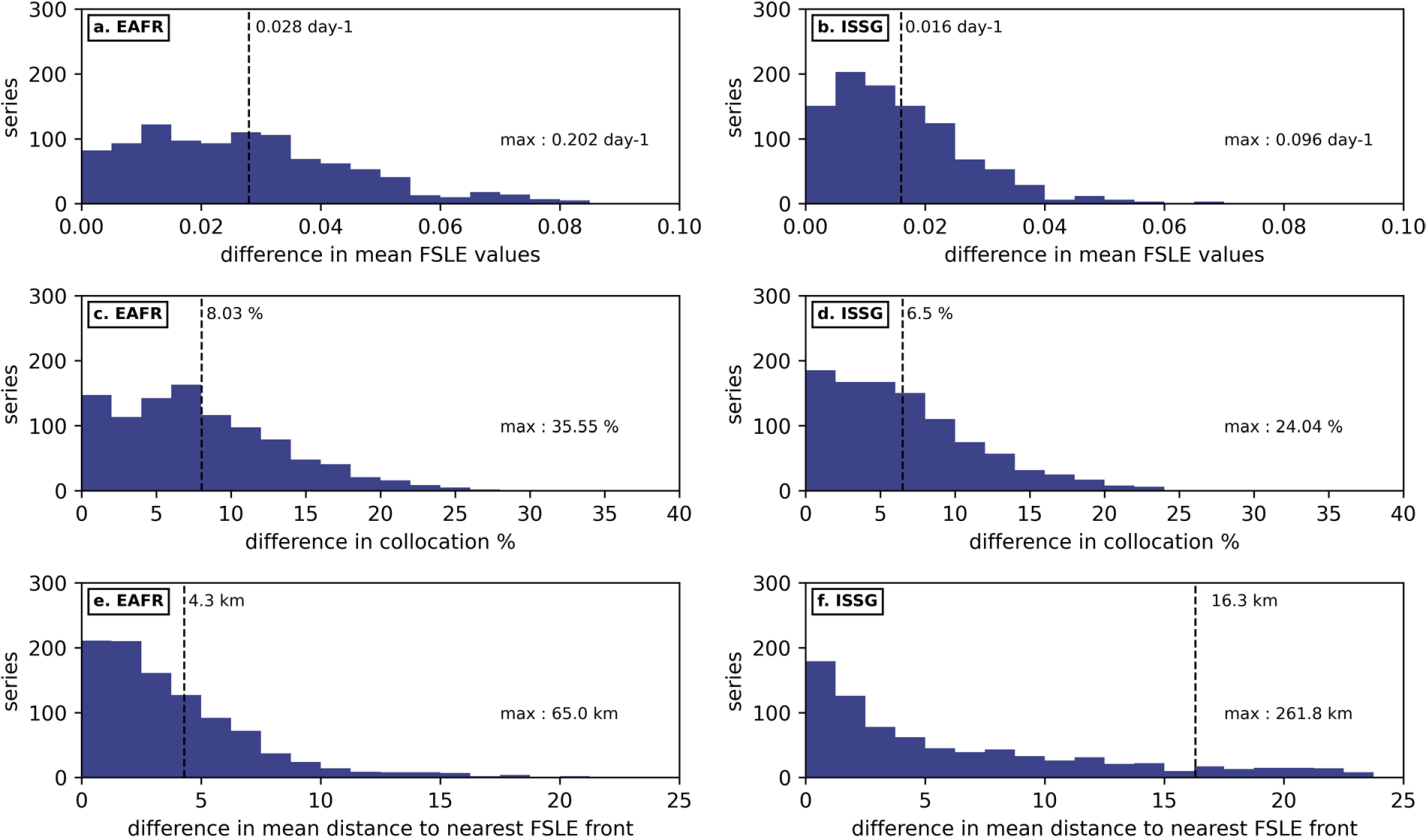
Distributions of the differences in mean FSLE values (**a, b**), collocation percentage (**c, d**) and distance to nearest front (**e, f**) between NOMAD^51^ and AVISO^44^ FSLE front fields for 1000 series of 100–200 randomly-generated points. We chose 2 Longhurst^48,49^ subregions to illustrate the region-specific sensitivity to spatial resolution: EAFR (**b**), ISSG (**c**). The vertical dotted lines mark the mean difference for all 1000 trajectories, weighted on the number of points per trajectory.

We thus obtain, for each series of randomly-generated points, a difference (between NOMAD^51^ and AVISO^44^ FSLE) for each metrics described above. These analyses were performed for both the EAFR and ISSG Longhurst subregions^48,49^ (Fig. 3) to illustrate the region-specific sensitivity to spatial resolution. The mean difference in FSLE values located under each randomly-generated point in the EAFR (resp. ISSG) subregion is of the order of 0.028 day^−1^ (resp. 0.016 day^−1^), and reaching up to 0.202 day^−1^ (resp. 0.096 day^−1^) (Fig. 8). The difference in collocation percentage is on average 8.03% in the EAFR and 6.5% in the ISSG subregions (Fig. 8), and going up to 35.55% and 24.04% respectively. Finally, the mean difference in distance to nearest front is smaller in EAFR at 4.3 km compared to 16.3 km in ISSG, likely due to the higher concentration of fronts in the Mozambique Channel^27^. Overall, the mean and maximum differences between fine and coarse FSLEs for those three metrics are non-negligible, especially in front-rich regions such as the Mozambique Channel and southeast of Madagascar, which will then reflect into any statistical comparison between biological observations and front occurrence. NOMAD’s^51^ finer spatial resolution is thus a significant added-value for high-resolution tracking of marine life-front interactions.

Finally, the backward FSLEs described in this paper were previously studied to analyze transport processes in the MedSea and were validated with trajectories of real drifters, as shown in Figs. 2b, 3. of Hernández-Carrasco and Orfila’s work^64^. Similar validation has been performed in the SWIO and is available upon request.

### Numerical diagnostics

#### Suitable day frequency

For each position in space (x, y) the frequency of “suitable” days (FSD) has been computed as follow:

$${\rm{FSD(x,\; y)}}=\frac{{\rm{NSD(x,\; y)}}}{{\rm{TND}}}\ast 100.$$

**Fig. 9:**
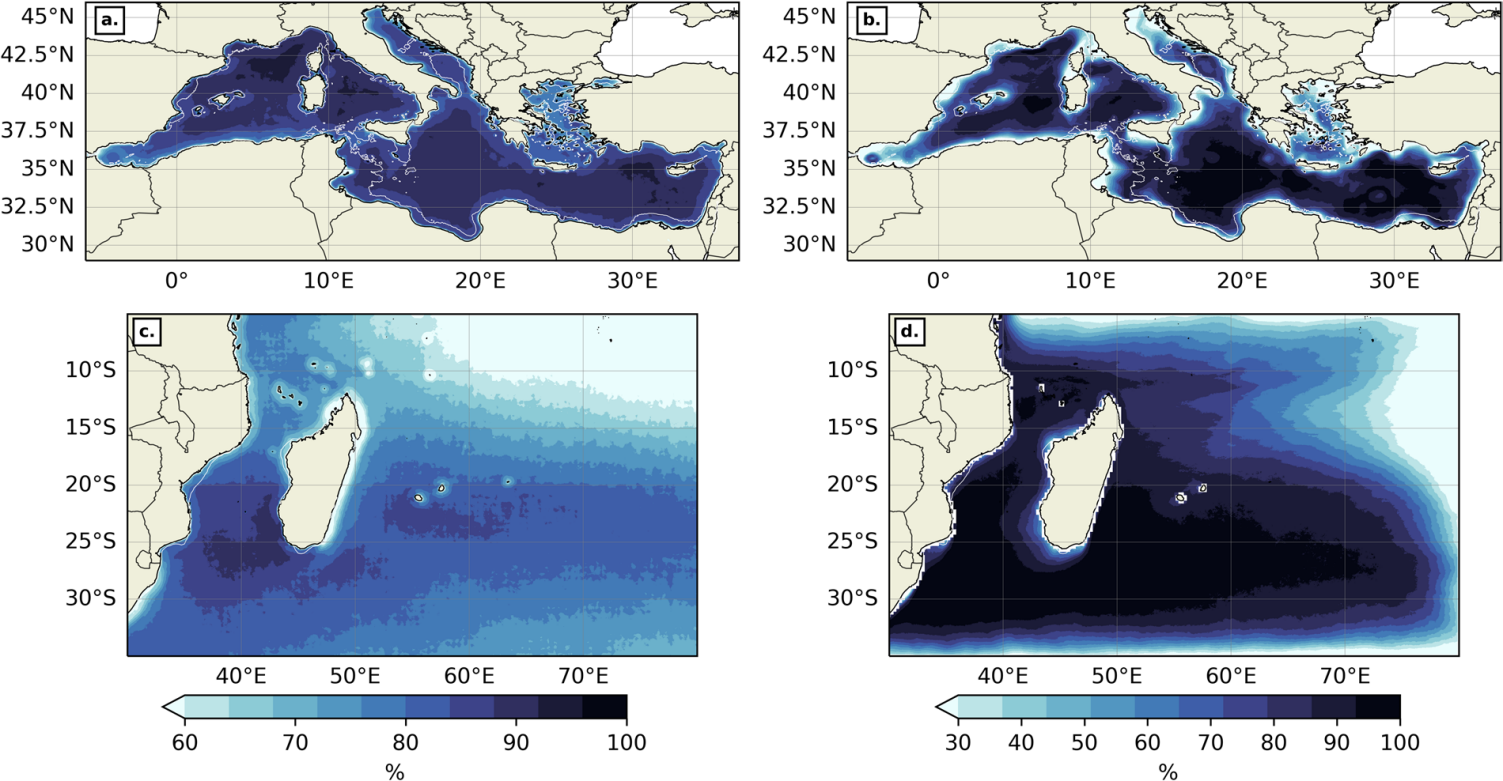
Frequency of suitable days for thermal gradients (**a, c**) and FSLEs (**b, d**) in the MedSea (**a, b**) and the SWIO (**c, d**). The background white contour shows the 200 m isobath. The colormap is from cmocean^80^.

Where NSD (x,y) is the number of “suitable” days at (x, y), i.e. when the flag is equal to 0, and TND is the total number of days in the whole dataset. The FSD for thermal gradients reveals which regions are more prone to cloudiness between 2003 and 2020 (Fig. 9a,c). In the MedSea, most of the open ocean presents a FSD above 85% thanks to the dominant clear sky typical of Mediterranean climate. The Mediterranean regions most covered by clouds are the western Alboran Sea, the northern Aegean Sea and more generally coastal areas. In the SWIO, most of the open ocean in the Mozambique Channel and east of Madagascar has a FSD above 80%. Continental shelves, Islands (the Comoros archipelago, Mayotte, La Réunion and Mauricius islands) and northeast of the South Equatorial Current are marked by a reduced FSD, but rarely fall below 50%. The FSD for the backward FSLE dataset reveals regions most accessible to advected ocean particles between 1994 and 2020 (Fig. 9b,d). In the MedSea, the FSD is lowest on continental shelves and western Alboran Sea but above 90% for most of the open ocean. In the SWIO, the FSD is above 90% in the Mozambique Channel and most of the western Indian Ocean. Lesser FSD can be found along the path of the South Equatorial Current, along the west Madagascar continental shelves and more generally at the border of the geographical domain. Overall both datasets and both geographical domains present a very good ratio of suitable pixel for front analysis.

#### Mean fields

**Fig. 10:**
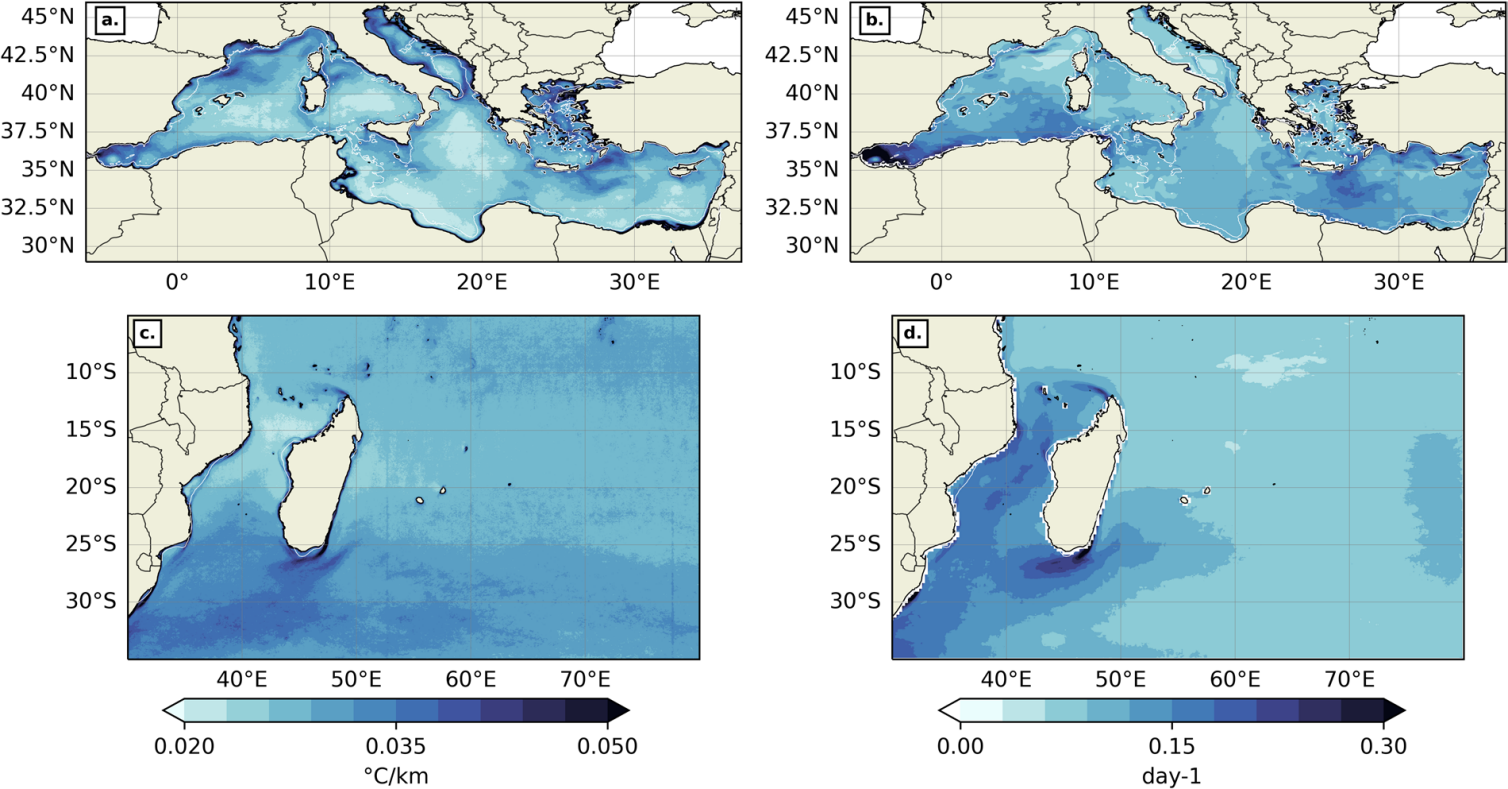
Time average for thermal gradients (**a, c**) and FSLEs (**b, d**) in the MedSea (**a, b**) and the SWIO (**c, d**). The background white contour shows the 200 m isobath. The colormap is from cmocean^80^.

Time average maps have been produced for both thermal gradients (Fig. 10a,c) and backward FSLE fields (Fig. 10b,d) in both geographical domains over their respective time period (2003–2020 for thermal gradients and 1994–2020 for backward FSLEs). Only the “suitable” pixels were counted when time-averaging. In the MedSea, thermal gradients are stronger on average along continental shelves and highlight specific regional features, such as the quasi-permanent eddies of the Alboran Sea, the Almeria-Oran front^65^, the Cartagena-Tenes Front^64^, and the Liguro-Catalan current. Time-averaged backward FSLE values are higher in the Alboran Sea, South of Sardinia, along the Liguro-Catalan current and in the Levantine Sea. In the SWIO, mean thermal gradients are most intense on continental shelves as well, but also in the wake of both Northeast and Southeast Madagascar currents (NEMC and SEMC) and more generally south of 25°S. These results were also shown from a 1/36° resolution realistic CROCO simulation of the Mozambique Channel^27^ and from Level 3 National Oceanic and Atmospheric Administration (NOAA) Advanced Very High Resolution Radiometer (AVHRR) SST data^11^. Time-averaged backward FSLE values are also stronger in the path of the NEMC and SEMC, but also at the side edges of the eddy train that characterizes the Mozambique Channel circulation^66,67^, and towards the Agulhas current^68^. These results are consistent with previous works on time-averaged thermal gradients^58^ and FSLEs in the MedSea^32,69^, but also in the SWIO^11,27^, thus validating our dataset for large time scales.

#### Front frequency

Another helpful metric to assess our dataset is the front frequency (FF), i.e. frequency of front days (amongst suitable days only) at a position (x, y), computed as follow:

$$\mathrm{FF(x,\; y)}=\frac{{\rm{SNFD(x,\; y)}}}{\mathrm{TNSD(x,\; y)}}\ast 100.$$

**Fig. 11:**
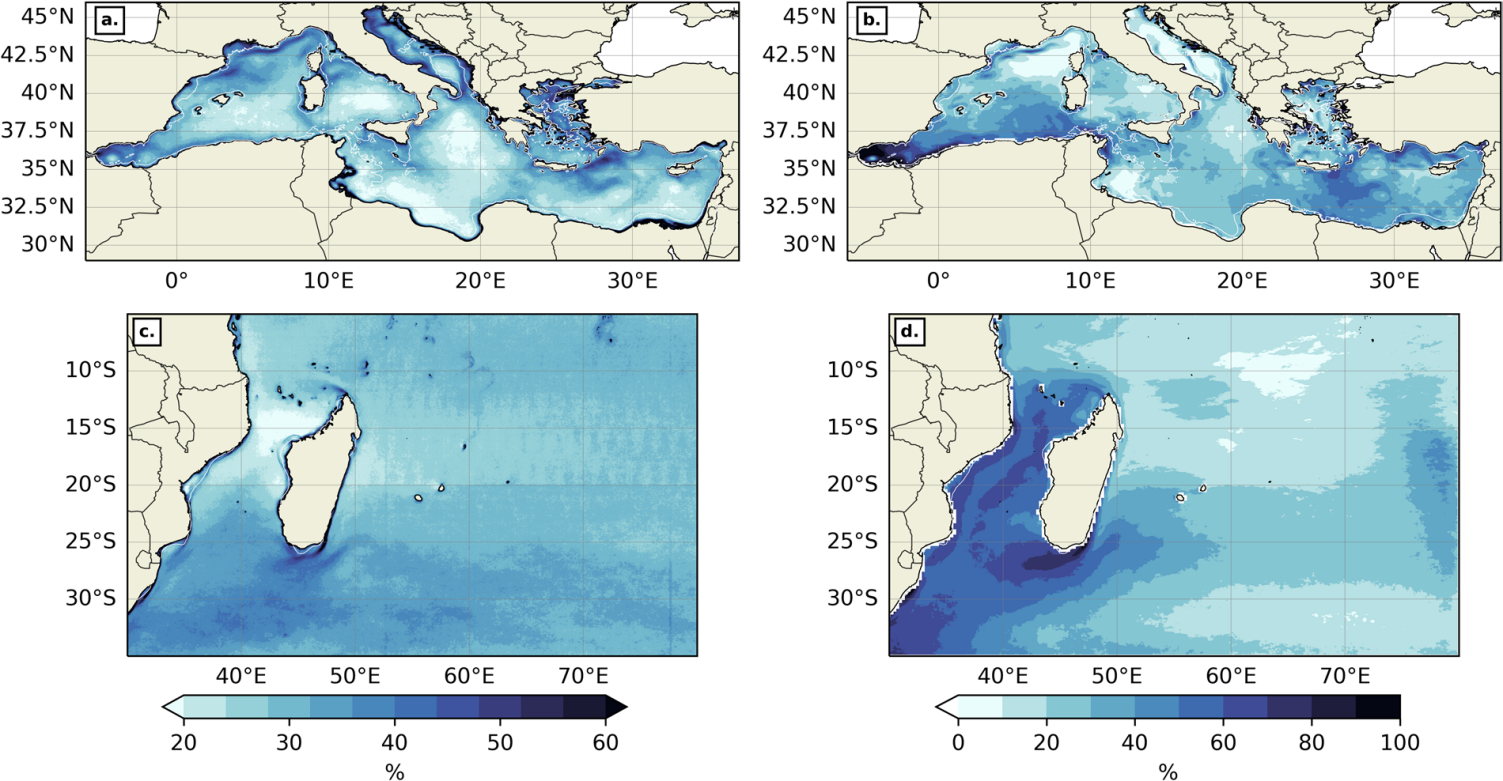
Front frequency for thermal gradients (**a, c**) and FSLEs (**b, d**) in the MedSea (**a, b**) and the SWIO (**c, d**). The background white contour shows the 200 m isobath. The colormap is from cmocean^80^.

Where SNFD (x, y) is the number of front days (amongst “suitable” days only) at (x, y), and TNSD (x, y) is the total number of “suitable” days at the same position. “Front days” correspond to days when thermal gradient of FSLE values at (x, y) are above a pre-determined 70^th^ percentile threshold. We chose to take whole region thresholds for both geographic domains. In the MedSea (resp. SWIO), the thermal gradient threshold was taken at 0.033 °C/km (resp. 0.035 °C/km), and the backward FSLE threshold at 0.123 day^−1^ (resp. 0.105 day^−1^). In both the MedSea and the SWIO, the spatial patterns of thermal (Fig. 11a,c) and FSLE-derived (Fig. 11b,d) front frequencies tend to overlap with the mean intensity maps of the same variables (Fig. 10). These results are consistent with the works of Nieblas *et al*.^11^ which used a different front detection method and a different SST product, and Sudre *et al*. when applying the BOA to numerically simulated temperature at different depths in the Mozambique Channel^27^.

#### About MUR SST analysis artefacts

Some artefacts are visible on thermal gradient mean and frequency figures (Figs. 9a,c, 10a,c, 11a,c). They are most visible in the SWIO, but can also be found in the MedSea. These criss-cross and square-like patterns are characteristics of satellite fingerprints and enhanced by MUR MRVA interpolations^46^. Similar satellite-SST-derived artefacts are also seen in Nieblas *et al*. Figure 8.D^11^. And indeed, the artefacts do not appear when the BOA^16^ is applied on numerical simulations instead of remotely-sensed SST^27^. More importantly, they are not visible in NOMAD^51^ thermal gradient snapshots (Fig. 2a,c) and therefore will not affect front-marine life interaction analysis. To conclude, since the artefacts are only produced after aggregating thermal gradients on long time-scales, the authors are confident they should not alter the reliability of the NOMAD^51^ thermal gradient product for biological use.

## Usage Notes

The oceaN frOnt for the Mediterranean seA and southwest inDian ocean dataset (NOMAD^51^) is a multi-decadal record of thermal gradients and backward FSLEs in the Southwest Indian Ocean and the Mediterranean Sea. The NOMAD^51^ distinguishes itself from existing alternatives^23,44^ by its higher temporal and spatial resolution and extent. NOMAD^51^ is suitable for a broad variety of applications where the detection of thermal fronts and attracting Lagrangian coherent structures in the MedSea and the SWIO is needed. These include the identification of spatial and temporal dynamics of thermal and dynamic fronts, along with multidecadal variability analyses. Additionally, the release of our front dataset makes the investigation of the relationship between fronts and marine life possible thanks to a high temporal and spatial resolution.

The provided dataset is divided into 4 folders as described in Table 1. Each folder contains yearly subfolders with daily netcdf files. The netcdf files can be read as is, as they directly provide direct thermal gradient or backward-in-time FSLE fields. We suggest to use Python language software with xarray and dask packages, which are best suited to handle large datasets and High Power Computing. NOMAD^51^ will be updated on a yearly basis.

### NOMAD visualization

The authors suggest using the quality flag provided in each dataset to select “suitable” pixels (i.e. flag = 0) before further front analysis. Then front detection is a simple thresholding of the field of interest as illustrated in Fig. 1. Subregion-specific thresholds are added as attributes of each netcdf file, but custom thresholding is also possible. Python xarray package makes filtering and thresholding of the data very intuitive with the function xarray.where. An example Jupyter Notebook for visualization of NOMAD^51^ data and the reproduction of Fig. 2 is available on Github: https://github.com/FlorianeSudre/NOMAD_notebooks/.

### Front collocation with biological data

NOMAD^51^ is primarily designed to study front-marine life interactions. To this end, the authors recommend using both thermal gradients and FSLE fields, and to interpolate them on the same grid, whose temporal and spatial scale is congruent with the behavior of the specie of interest. Temporal degradation of the dataset is easily performed by xarray.Dataset.resample while spatial regridding can be done with tools such as the Climate Data Operator (CDO) or with the Python package xgcm used in tandem with xarray). The authors recommend comparing quantitatively and qualitatively BOA outputs solely between same-grid products. For example, one can compare thermal gradient magnitudes and patterns within the entire NOMAD^51^ thermal gradient dataset, as the SST field comes from a single product. However, while qualitative comparison (i.e. spatial patterns) is possible among thermal gradients originating from different SST fields, a quantitative comparison would require to determine a suitable threshold for each product since BOA algorithm is sensitive to the grid resolution of the input data. Attention should also be brought to the fact that thermal gradients and FSLEs do not have NaNs (where the pixel is deemed “unsuitable”, i.e. where the quality flag is not equal to 0) at the same time and place. NaNs appear in FSLE fields near domain boundaries and in coastal areas, while NaNs in thermal gradient fields depend on cloud cover (Fig. 9). The difference in NaN coverage in both dataset should be taken into account when pairing with biological data to avoid any additional bias due to suitable day frequency.

### Other possible uses for NOMAD

Beside the study of front-marine life interactions^10^, investigating trophic relationships^70^ and the accumulation of fish larvae^71^, NOMAD^51^ could also be employed to monitor marine pollutants such as microplastics^72,73^ and oil spills^74^ along fronts. One could also examine relationships between surface thermal fronts and low-level winds at submesoscales from Radar Scatterometer data; as it has only been done at mesoscales so far^75–77^. Finally, following the recent work of Roman-Stork *et al*.^78^, it would be interesting to explore new blended metrics based on both SST fronts and FSLEs and their co-variability with key biogeochemical elements (such as Chl-a, pH, nitrates, phosphates) to inform on where oceanic fronts are more likely to impact nutrient cycling in the upper and interior ocean^79^.

## Data Availability

The code we used to produce thermal gradients with the BOA is available in R on Github thanks to Benjamin Galuardi (available on https://github.com/galuardi/boaR, accessed on May 19th, 2022). The code to produce the backward FSLEs belongs to Ismael Hernández-Carrasco, was extensively described in Hernández-Carrasco *et al*.^34^, and is available upon request. An example jupyter notebook for front visualization is available on a github repository (https://github.com/FlorianeSudre/NOMAD_notebooks).
